# Limb Length Inequality in Patients After Primary Total Hip Arthroplasty: Analysis of Radiological Assessment and Influencing Risk Factors Based on a District General Hospital Experience of 338 Cases

**DOI:** 10.7759/cureus.19986

**Published:** 2021-11-29

**Authors:** Amr Abouelela, Islam Mubark, Mohamed Nagy, Jamie Hind, Nithish Jayakumar, Neil Ashwood, Francesco Bindi

**Affiliations:** 1 Trauma and Orthopaedics, University Hospitals of Derby and Burton National Health Service Foundation Trust, Derby, GBR; 2 Trauma and Orthopaedics, Cairo University Hospitals, Cairo, EGY; 3 Trauma and Orthopaedics, Walsall Manor Hospital, Burton-on-Trent, GBR; 4 Orthopaedics, University Hospitals of Derby and Burton National Health Service Foundation Trust, Derby, GBR

**Keywords:** body mass index, preoperative templating, anesthesia, radiographic measurement, total hip arthroplasty, limb length inequality

## Abstract

Background and objective

Limb length inequality (LLI) is a frequent and recurring issue after total hip arthroplasty (THA). It is often a source of patient dissatisfaction and litigation. This study reviewed the incidence of LLI in a UK District General Hospital in light of published evidence and identified the preoperative and intraoperative risk factors for LLI.

Methods

This was a retrospective study involving 380 consecutive unilateral primary total hip replacements over a period of 12 months. Patient demographics, clinical, radiological, and operative details were collected from the National Joint Registry (NJR) database and hospital records. The limb length was measured radiologically [OrthoView Workstation^TM ^(Materialise UK, Southampton, UK)], pre- and postoperatively, by two authors. They assessed the vertical distance between the intra-acetabular teardrop line and the medial apex of the lesser trochanters. After excluding complex primary, revision cases, tilted X-rays, and hip replacement for trauma patients, 338 cases were included in the final analysis.

Results

The mean postoperative LLI was 2.7 mm with a standard deviation (SD) of 6.56 mm. Only 5.3% of patients had LLI >15 mm. None of the studied variables showed a statistically significant correlation with LLI. Even with the apparent difference in the mean LLI between templating and not templating before surgery (2.19 vs. 3.53), the p-value was 0.06, which was below the level of statistical significance. There was a weakly positive Pearson correlation between body mass index (BMI) and the incidence of lengthening of the limb.

Conclusion

The cause of LLI after THA is multifactorial. No single factor can be singled out as the most significant contributor to this complication.

## Introduction

Limb length inequality (LLI) after total hip arthroplasty (THA) is one of the most common causes of litigation in orthopedic surgery and can unfavorably affect an otherwise satisfactory outcome [1]. The NHS Litigation Authority has reported that THA accounted for 1,004 claims from 1995 to 2010, at the cost of over £41.5 million. Of these, 100 claims were related to LLI. The mean pay-out per case was £84,000, and the highest was £595,000 [2].

Konyves et al. have reported a study in which the mean Oxford Hip Score in limb-lengthened patients was 27% worse than the rest of the population at three months and 18% worse after 12 months. Patient dissatisfaction was attributed to various consequences of limb lengthening during the initial postoperative period, as the patients started walking with a tilted pelvis, thereby inducing secondary symptoms such as back pain and sciatica, neuritis, gait disorders, recurrent dislocation, and early loosening of components [3,4].

Balancing the limb length during THA, therefore, remains a key challenge for arthroplasty surgeons, and practitioners who have adopted multiple different pre- and intraoperative techniques to avoid any LLI. The preoperative measures include history taking and clinical examination to identify any pre-existing structural LLI or other risk factors. These factors include congenital dysplasia, growth plate problems, previous hip surgery, fractures, or infections [5]. Surgeons also use preoperative electronic templates to help determine the center of rotation, select implant size, and position and guide the femoral osteotomy level [6].

Intraoperative measures mainly depend on assessing soft tissue tension and limb length using different fixed reference points [6]. However, robust evidence for the beneficial effects of each of these measures on LLI is still lacking.

A review of the literature suggests that the incidence of LLI after primary THA varies from 1% to 27%, and the mean discrepancy ranges from 3 mm to 17 mm [7]. There is a broad consensus in the literature that a residual LLI of less than or equal to 10 mm on anteroposterior (AP) pelvic radiographs is clinically acceptable and well-tolerated by most patients [8-10]. However, there is no agreement on an upper cut-off point that would be considered unacceptable [11].

The British Hip Society (BHS) conducted an interesting survey of 394 of its members regarding the acceptable ranges of LLI and its effect on the outcomes of THA. Most surgeons (89%) felt that up to 15 mm of LLI after primary uncomplicated THA was always tolerable and constituted a satisfactory outcome. Further, 90% believed that an LLI of more than 22.74 mm was never tolerable. The survey results did not provide concrete values for acceptable cut-off ranges for the LLI. However, this was an unprecedented study that attempted to produce a consensus on the matter [6].

There is still a lack of evidence regarding LLI in District General Hospitals and regarding the effect of body mass index (BMI) and type of anesthesia on limb length balancing. In light of this, the purpose of this study was to evaluate the practice at our institution against the published literature by measuring the LLI in THA on radiographs pre- and postoperatively and assessing the effect of poorly studied risk factors for LLI, such as fixation method, the type of anesthesia, type of approach, and BMI. We conducted a retrospective analysis of LLI in primary unilateral THA patients who underwent arthroplasty surgery at our institution over a 12-month period from June 2018 through June 2019.

## Materials and methods

Study setting

Our institution is a busy District General Hospital serving a population of 360,000 people. Nine fellowship-trained orthopedic consultants provide hip and knee arthroplasty services in our department. This study was a retrospective analysis of 380 patients who underwent unilateral primary THA at our institution over a 12-month period from June 2018 to June 2019.

Data source

The patients were identified through the National Joint Registry (NJR) database records kept at the institution. Relevant patient demographics and operative notes were obtained from the electronic patient records.

Data collection and analysis

Pre- and postoperative AP pelvic radiographs were accessed through our digital imaging system. They were taken in a non-weight-bearing mode with the legs in 15-20 degrees of internal rotation so that the femoral head and neck were parallel to the radiographic cassette to facilitate templating and accurate measurements [12]. 

The exclusion criteria were as follows: complex primary hip arthroplasty (e.g., dysplastic hip, ankylosed hip, fractures about the hip, protrusio acetabuli, neuromuscular conditions, skeletal dysplasia, previous bony procedures on the hip [13]), pre-existing LLI in patients not attributable to hip pathology, revision cases, tilted X-rays, and fractured neck of femur patients where no prior X-rays were available to measure the preoperative limb length discrepancy. A total of 338 cases met the eligibility criteria and were included in the study.

Pre- and postoperative radiographs were assessed by two of the authors. They independently measured the pre- and postoperative leg length discrepancies using OrthoView Workstation^TM^ (Materialise UK, Southampton, UK) version: 6.0.12_WK. To ensure accurate calculations, radiographic magnification factors were calculated from the postoperative radiographs using the known sizes of prosthesis head, in cemented cups, and cup sizes in uncemented cups. The mean magnification was 124.4 ±2.77, which was then applied on pre- and postoperative radiographs for LLI calculation.

**Figure 1:**
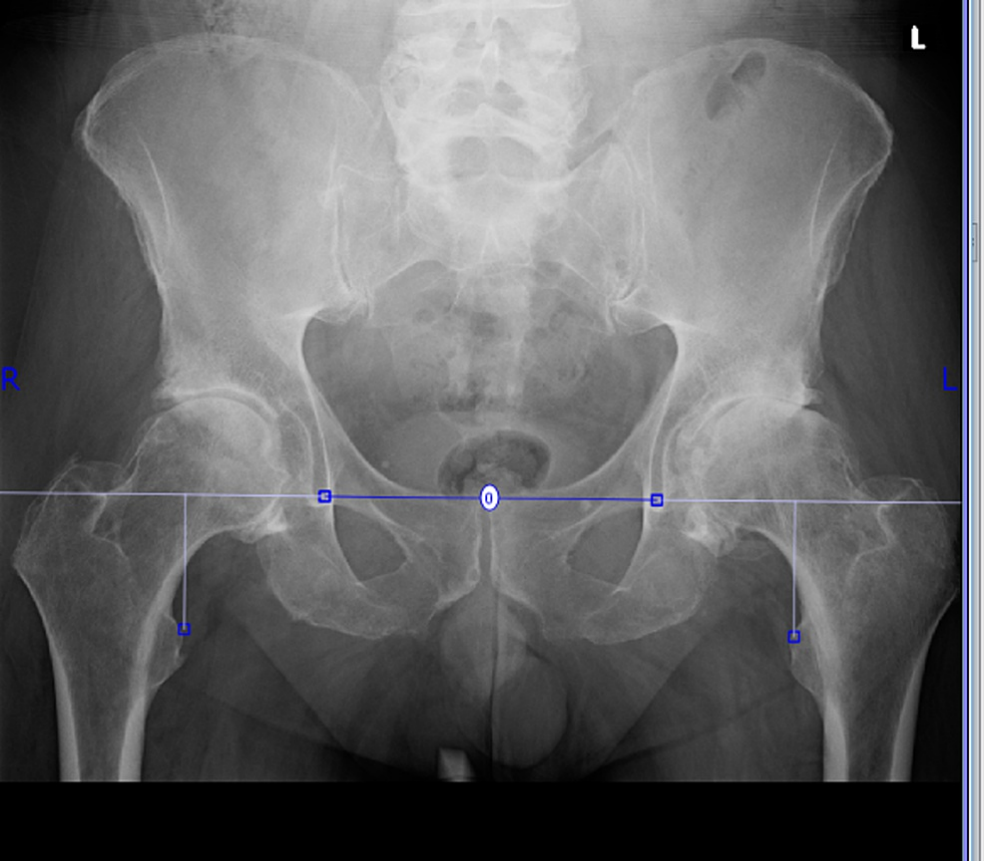
A preoperative anteroposterior pelvis radiograph of THA The image shows the limb length measurement as the vertical distance between the inter-acetabular teardrop line and the most prominent medial point of the lesser trochanter using the OrthoView Workstation^TM^ THA: total hip arthroplasty

Limb length was measured as the vertical distance between the inter-acetabular teardrop line and the most prominent medial point of the lesser trochanter (Figure 1) [14].

LLI (change in length) has been reported as either a positive value to indicate lengthening of the operated leg or a negative value to indicate shortening. The mean of two results was used for analysis.

Patient demographics (gender, age, BMI) and surgical parameters (operating surgeon, approach, type of anesthesia, and preoperative templating) were collected. The name and grade of the operating surgeon were retrieved from the operative notes and the NJR database and blinded to the authors to minimize any bias during the measurement of the LLI on radiographs. Calculations of averages and standard deviations were done using MS Excel 2010 (Microsoft Corp., Redmond, WA). Then, a two-sample paired t-test was performed, assigning the associated p-value for each category. The SPSS Statistics software version 16.0 (IBM, Armonk, NY) was used to assess the variations in measurements and possible risk factors.

## Results

Out of 338 patients, the majority were female (59.8%). Patient demographics are presented in Table 1. The mean age was 70.3 years (range: 39-96 years), with an average BMI of 29.01 kg/m^2^ (range: 18-45 kg/m^2^).

**Table 1:** Demographic, descriptive, and postoperative statistics of the patients BMI: body mass index; LLI: limb length inequality

Variables	Lower range	Upper range	Mean	Standard deviation
Age in years	39	96	70.63	10.122
BMI in kg/m^2^	18	45	29.01	4.509
LLI preoperative in mm	−21	−12	−2.85	5.008
LLI postoperative in mm	−17	18	2.74	6.559
Change in limb length in mm	−16	28	5.59	7.013

The majority of the patients (84%) had spinal anesthesia (n=284) and 16% (n=54) had general anesthesia. Most patients (70%) were operated on through the anterolateral approach, while the remaining were through a posterior approach. Preoperative templating was performed and recorded for 59% of the patients. There was no record in the remaining cases. Cemented implants were used in 45.9% of patients, followed by hybrid in 36.7% and uncemented in 17.5%.

LLI 

The mean preoperative LLI was −2.85 mm (range: −21 to −12 mm), where the operated side was always shorter than the non-arthritic side. The mean postoperative LLI as measured on the AP radiograph was 2.74 mm (range: −17 to 18 mm) with a standard deviation (SD) of 6.56 mm. The proportion of LLIs over 15 mm was only 5.3%. Our study mean was much better than the published means in the literature, which varied from 3 to 17 mm [7].

The relationship between LLI and different variables was studied to determine the possible risk factors and their influence on balancing limb lengths. When preoperative templating was performed, the mean LLI was 2.19 mm vs. 3.53 mm in cases where no evidence of templating was recorded. However, this difference was not statistically significant (p=0.06).

There were no outliers among the nine operating surgeons with very close means for the LLI. Similarly, with the type of anesthesia, despite a lower mean in regional anesthesia than in general, it was not statistically significant. The type of fixation and approach showed no influence on the overall limb length balancing. There was a weakly positive Pearson correlation between BMI and incidence of limb lengthening (Table 2). 

**Table 2:** Pearson correlation between BMI and LLD incidence BMI: body mass index; LLD: leg length discrepancy

		BMI	LLD
BMI	Pearson correlation	1	0.068
	Sig. (2-tailed)		0.215
	N	338	338
LLD	Pearson correlation	0.068	1
	Sig. (2-tailed)	0.215	
	N	338	338

## Discussion

The growth in our aging population has resulted in an exponential increase in the number of THAs performed. The number of patients dissatisfied with the THA results has also risen. One of the main causes of dissatisfaction after THA is LLI, mainly lengthening. This appears to be reported more frequently these days than in the past [15]. According to the NHS Litigation Authority report, from 1995 to 2010, litigation related to LLI comprised about 10% of all THA litigations [2]. Balancing the limb length without compromising hip stability remains one of the hardest intraoperative challenges [10,16].

Many techniques have been advocated in the literature to help the surgeon achieve the desired limb length after the hip replacement: perioperative templating, the use of intraoperative pelvic or femoral markers for reference, and computer-assisted techniques, or navigation, to measure limb lengths precisely [17]. 

It is also very important that the surgeon defines the preoperative LLI clinically and radiologically to aid surgical planning. We relied on the non-weight-bearing plain pelvic radiographs, which were standardized by positioning the legs at 15-20 degrees of internal rotation so that the femoral head and neck were parallel to the radiographic cassette to ensure accurate measurements [12]. Plain radiographs are the gold standard of investigation for measuring LLI. Their validity and reliability have been investigated and found clinically acceptable [18]. Scanograms of the limb have been used as an alternative to plain radiographs to assess LLI introduced by other segments in the lower limb [19], although they are not performed as part of the routine follow-up. CT scans offer a more precise measurement of LLI; however, they have a limited role owing to the added radiation exposure and costs [20].

Regarding templating, the difference between the mean LLI (2.19 mm) in cases with prior templating compared to 3.53 mm in those without prior templating did not quite reach statistical significance (p=0.06). This may be a reflection of the relatively small number of cases in the study. In a recent systemic review, preoperative templating resulted in a significant increase in the accuracy of THA [21]. However, the exclusive dependence on templating resulted in correct size-matching in only up to 60% of cases in other previous studies [22].

With respect to the type of anesthesia, general anesthesia in THA has generally fallen out of favor primarily due to the lower risks of postoperative complications with regional anesthesia [23-26]. With regional anesthesia, there is an overall soft tissue laxity that facilitates surgical exposure [24-27]. The intraoperative soft tissue tension assessment by the surgeon plays an important role in their choice of implant size and affects the overall limb lengthening. The surgeons rely predominantly on intraoperative tests that depend on the soft tissue tension, such as the Shuck or Kick test [28]. A survey conducted by the BHS investigated the intraoperative techniques used by its members to minimize LLI after THA. Around 80% of the respondents answered that they relied on the Shuck test and generally felt happy to balance the limb length intraoperatively. Further, 62.2% relied on the Kick test [6].

In our study, most of the patients (84%, n=284) had regional anesthesia while only 16% (n=54) had general anesthesia. The difference was not statistically significant. Sathappan et al. [7] reported different results after retrospectively studying 132 THA patients equally distributed among anesthesia types. LLI over 10 mm was observed in 87% of patients who received regional anesthesia as opposed to 47.6% of the patients who had general anesthesia (p<0.001). They recommended using additional intraoperative measures of assessing hip stability and detailed preoperative templating when operating on patients under regional anesthesia to avoid LLI [7]. 

**Figure 2:**
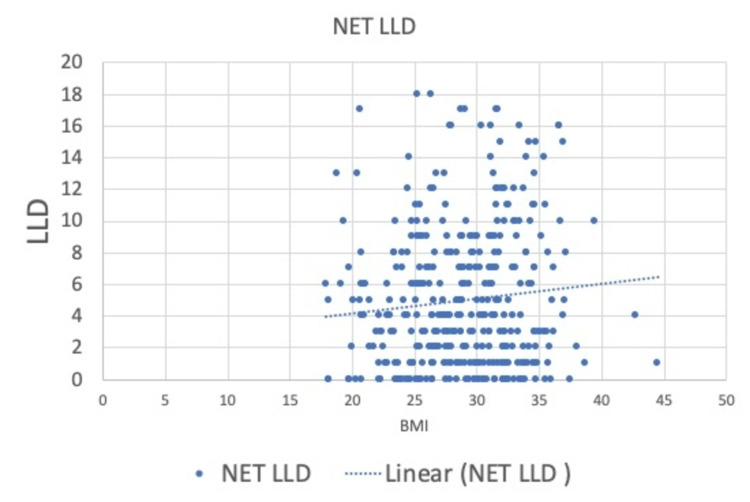
Pearson correlation between BMI and LLD incidence BMI: body mass index; LLD: leg length discrepancy

When we analyzed the effect of the BMI on the overall LLI, there was a weakly positive Pearson correlation between BMI and incidence of lengthening of the limb (Figure 2). The mean BMI in our study was 29.01 Kg/m^2^ (range: 18-45 Kg/m^2^) Al-Amiry et al. [29] reported that increased BMI showed a negative effect on the restoration of post-THA leg length but not on the restoration of femoral offset or positioning of the acetabular cup. They conducted a prospective study on a smaller cohort of patients (n=213) with a lower mean BMI of 27.7 (SD: 4.5). They studied the effect of BMI on LLI, femoral offset, and acetabular component inclination, and anteversion using postoperative radiographs. They reported BMI as the only factor that affected LLI after multivariable logistic regression analysis. Increased BMI increased the risk of LLI (OR: 1.14, 95% CI: 1.04-1.25) [29].

The present study has some limitations. Plain radiographs were the only parameter used for measuring LLI. Although their validity and reliability have been confirmed [18], they might underestimate the LLI in cases of subtle hip malposition and fixed flexion deformity. The limited number of patients having general anesthesia compared to those who had regional anesthesia entails a limitation on the overall statistical analysis comparing both groups. Finally, the limited number of patients with high BMI in the study would have affected the statistical analysis of the relationship between LLI and BMI.

## Conclusions

Our results for postoperative LLI compare very favorably with those in the literature, with a mean lengthening of 2.74 mm documented in our study group. The cause of LLI after THA is multifactorial. No single patient-, surgical-, or implant-related factor significantly influences LLI. Although preoperative templating did not achieve a statistically significant benefit in our study, it was close to doing so (p=0.06), and we would recommend its use in helping to reduce the risk of LLI. Patients with a high BMI are at a higher risk of postoperative LLI.
